# Molecular docking studies on InhA, MabA and PanK enzymes from *Mycobacterium tuberculosis* of ellagic acid derivatives from *Ludwigia adscendens* and *Trewia nudiflora*

**DOI:** 10.1186/s40203-015-0014-1

**Published:** 2015-12-08

**Authors:** Jamil A. Shilpi, Mohammad Tuhin Ali, Sanjib Saha, Shihab Hasan, Alexander I. Gray, Véronique Seidel

**Affiliations:** Natural Products Research Laboratories, Strathclyde Institute of Pharmacy and Biomedical Sciences, University of Strathclyde, Glasgow, UK; Pharmacy Discipline, Life Science School, Khulna University, Khulna, Bangladesh; Department of Biochemistry and Molecular Biology, University of Dhaka, Dhaka, Bangladesh; Bioinformatics Laboratory, QIMR Berghofer Medical Research Institute, Brisbane, Australia; School of Medicine, University of Queensland, Brisbane, Australia

**Keywords:** *Mycobacterium tuberculosis*, Ellagic acid derivatives, 2-*trans*-enoyl-ACP reductase (InhA), β-ketoacyl-ACP reductase (MabA), Pantothenate kinase (PanK), *Ludwigia adscendens*, *Trewia nudiflora*

## Abstract

**Purpose:**

There is an urgent need to discover and develop new drugs to combat *Mycobacterium tuberculosis*, the causative agent of tuberculosis (TB) in humans. In recent years, there has been a renewed interest in the discovery of new anti-TB agents from natural sources. In the present investigation, molecular docking studies were carried out on two ellagic acid derivatives, namely pteleoellagic acid (**1**) isolated from *Ludwigia adscendens*, and 3,3′-di-*O*-methyl ellagic acid 4-*O*-*α*-rhamnopyranoside (**2**) isolated from *Trewia nudiflora*, to investigate their binding to two enzymes involved in *M. tuberculosis* cell wall biogenesis, namely 2-*trans*-enoyl-ACP reductase (InhA) and β-ketoacyl-ACP reductase (MabA), and to pantothenate kinase (PanK type I) involved in the biosynthesis of coenzyme A, essential for the growth of *M. tuberculosis*.

**Methods:**

Molecular docking experiments were performed using AutoDock Vina.

The crystal structures of InhA, MabA and PanK were retrieved from the RCSB Protein Data Bank (PDB). Isonicotinic-acyl-NADH for InhA and MabA, and triazole inhibitory compound for PanK, were used as references.

**Results:**

Pteleoellagic acid showed a high docking score, estimated binding free energy of −9.4 kcal/mol, for the MabA enzyme comparable to the reference compound isonicotinic-acyl-NADH.

**Conclusions:**

Knowledge on the molecular interactions of ellagic acid derivatives with essential *M. tuberculosis* targets could prove a useful tool for the design and development of future anti-TB drugs.

**Electronic supplementary material:**

The online version of this article (doi:10.1186/s40203-015-0014-1) contains supplementary material, which is available to authorized users.

## Background

*Mycobacterium tuberculosis*, the causative agent of tuberculosis (TB) in humans, is the leading bacterial killer worldwide. It led to 1.5 million deaths in 2013. TB rates are particularly high in developing countries where, with HIV/AIDS and malaria, it creates a huge burden on healthcare systems. The current recommended treatment for TB involves a prolonged course of a combination of antibiotics with toxic side-effects and is associated with poor patient compliance. This has led to the emergence of multi-drug resistant (MDR) and extensively-drug resistant (XDR) strains of *M. tuberculosis* (WHO 2014). The treatment of MDR-TB requires expensive second-line drugs whilst XDR-TB is often incurable. The number of anti-TB drugs currently in the pipeline is insufficient to address this major health challenge. Therefore, there is an urgent need to discover and develop new and efficient drugs against TB (Zumla et al. 2013). In addition to that, new antimycobacterial agents are needed to improve the treatment of chronic infections caused by non-tuberculous mycobacteria which have become difficult to treat (Johnson and Odell 2014).

Several key enzymes involved in *M. tuberculosis* cell wall biogenesis and physiological functions have become attractive targets for the design of novel anti-TB agents (Jackson et al. 2013). Two of the target proteins of interest in this study, namely 2-*trans*-enoyl-ACP reductase (InhA) and β-ketoacyl-ACP reductase (MabA), belong to the type-II fatty acid elongation system (FAS-II). The latter is a complex group of enzymes responsible for the production of very long chain fatty acid derivatives that are key precursors to mycolic acids, the main constituents of *M. tuberculosis* cell wall (Marrakchi et al. 2000, 2002; Takayama et al. 2005). Both enzymes are functionally and structurally-related. They display the same specificity for long chain substrates and are similarly inhibited by the front-line anti-TB drug isoniazid (Quemard et al. 1995; Marrakchi et al. 2000, 2002; Ducasse-Cabanot et al. 2004). Another target for the development of novel anti-TB drugs is the enzyme pantothenate kinase (PanK, type I) involved in the biosynthesis of the cofactor Coenzyme A (CoA) from pantothenic acid, which is essential for the growth of *M. tuberculosis* (Bjorkelid et al. 2013).

Natural sources represent a vast reservoir of chemically-diverse molecules which can provide new templates for drug design. There has been a renewed interest in recent years in the discovery of antimycobacterial/anti-TB agents from natural sources (Guzman et al. 2012; Dashti et al. 2014; Santhosh and Suriyanarayanan 2014). Among these natural products, ellagic acid derivatives are known to interfere with mycolic acid biosynthesis (Kondo et al. 1979). *Ludwigia adscendens* and *Trewia nudiflora* were selected as part of a project on the discovery of antimicrobial products from Bangladeshi medicinal plants. We previously reported on the phytochemical investigation of *L. adscendens,* leading to the isolation of pteleoellagic acid (**1**) (Shilpi et al. 2010). In this work, we report on the isolation of compound **2** from *T. nudiflora* and on molecular docking studies of **1** and **2** on InhA, MabA and PanK enzymes from *M. tuberculosis*.

## Methods

### Isolation and characterisation of compound 2

The plant *Trewia nudiflora* L. was collected in Rajshahi, Bangladesh, in May 2006 and a voucher specimen (DACB 34427) was deposited at the Bangladesh National Herbarium. The air-dried powdered stem bark (1.1 kg) was subjected to accelerated solvent extraction using an ASE 100® system (Dionex, UK) successively with *n*-hexane, ethyl acetate and methanol. Operating conditions comprised of four static cycles (one cycle = 8 min); oven temperature 100 °C, flush volume 60 %, purge time 150 s, pressure 1400–1500 psi. The methanol extract was successively partitioned with *n*-hexane, ethyl acetate and butanol. The butanol phase was further fractionated by vacuum liquid chromatography using silica gel 60H (VWR International, UK). The fraction eluted with 35 % methanol in ethyle acetate was chromatographed on a C-18 silica column (10 g, Phenomenex, UK) using a Flash Master Personal® system (Biotage, UK). Elution with 100 % water, followed by gradual increases of acetone, yielded compound (**2**) (46 mg) as a light brown amorphous solid. Characterisation work was performed by a combination of mass spectrometry and ^1^H and ^13^C nuclear magnetic resonance spectroscopy experiments, acquired on a ThermoFinnigan LCQ- Orbitrap and a JEOL- 400 Lambda Delta instrument, respectively.

### Molecular docking studies

#### Ligand and protein preparation

**Fig. 1:**
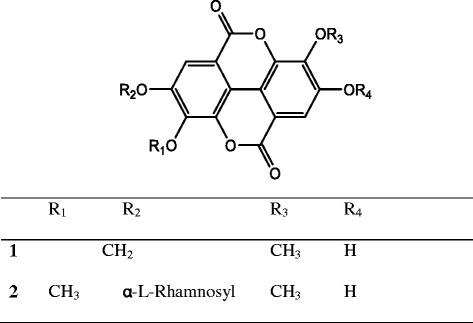
Chemical structures of ellagic acid derivatives

ChemBio3D Ultra 12.0 (www.cambridgesoft.com) was used to draw the structures of compounds **1** and **2** (Fig. 1), optimise ligand geometry and run MM2 energy minimisation of the 3D structures (Allinger 1977). The structures of the experimental inhibitors, isonicotinic-acyl-NADH for InhA and MabA, and triazole inhibitory compound for PanK, were retrieved from the respective protein crystal structures (PDB ID: 1ZID and PDB ID: 4BFT, respectively). All file conversions required for the docking study were performed using the open source chemical toolbox Open Babel version 2.3.2 (www.openbabel.org) (O’Boyle et al. 2011). All rotatable bonds present on the ligands were treated as non-rotatable to perform the rigid docking. The Gasteiger charge calculation method was used and partial charges were added to the ligand atoms prior to docking (Gasteiger and Marsili 1980). The crystal structures of InhA (PDB ID: 1BVR), MabA (PDB ID: 1UZN) and PanK (PDB ID: 3AF3) were retrieved from the RCSB Protein Data Bank (PDB) (www.rcsb.org/pdb/home/home.do). All water molecules and hetero atoms were removed from the crystal structures by using PyMOL molecular graphic system, version 1.5.0.3 (www.pymol.org).

#### Identification of binding site residues

The binding site residues for InhA and MabA were identified from previous studies (Rozwarski et al. 1999; Marrakchi et al. 2000; Rosado et al. 2012). The active site residues of PanK were retrieved from the analysis of the crystal structures of PanK in complex with pantothenate (PDB ID: 3AF3) and the triazole inhibitory compound (PDB ID: 4BFT) and its sequence annotation available in the Uniport database (Accession number: P9WPA7).

#### Grid box preparation and docking

Docking experiments were performed with compound **1**, **2** and the experimental control inhibitors against InhA, MabA and PanK proteins. Grid box parameters (Table 1) were set by using AutoDock Tools (ADT), a free graphic user interface of MGL software packages (version 1.5.6rc3) (Morris et al. 2009). The molecular docking program AutoDock Vina (version 1.1.2) (Trott and Olson 2010) was employed to perform the docking experiment. The Lamarckian Genetic Algorithm was used during the docking process to explore the best conformational space for the ligand with a population size of 150 individuals. The maximum numbers of generation and evaluation were set at 27,000 and 2,500,000, respectively. Other parameters were set as default (Table 1).

**Table 1:** Binding site residues and grid box parameters selected for the target enzymes

Protein name	Binding site residues	Centre grid box (points)	Size (points)	Spacing (Å)
InhA	Met103, Phe149, Met155, Tyr158, Met161, Ala198, Met199, Ala201, Ile202, Leu207, Ile215, Leu218, and Thr196.	12.832 × 16.388 × 6.306	20 × 20 × 20	1.0
MabA	Gly22, Asn24, Ile27, Arg47, Asp61, Val62, Gly90, Asn88, Ser140, Ile138, Gly139, Tyr153, Ile186, and Lys157.	3.561 × 17.242 × 11.951	22 × 22 × 22	1.0
PanK	Gly97, Ser98, Val99, Ala100, Val101, Gly102, Lys103, Ser104, His179, Tyr235, Arg238, Met242, Asn277	−40.278 × 34.674 × −5.52	20 × 20 × 20	1.0

## Results and discussion

**Fig. 2:**
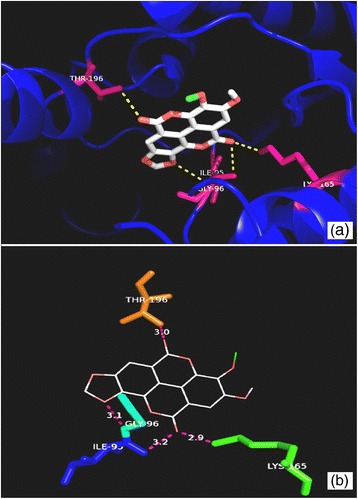
Molecular interactions between compound **1** and InhA. **a** Docked pose of **1**in the InhA binding site. The residues which interact with **1** are marked in a *hot pink colour*. **b** Interactions between **1** and InhA with the H-bond distances generated by PyMOL. *Dashed lines* represent the H-bonds

**Fig. 3:**
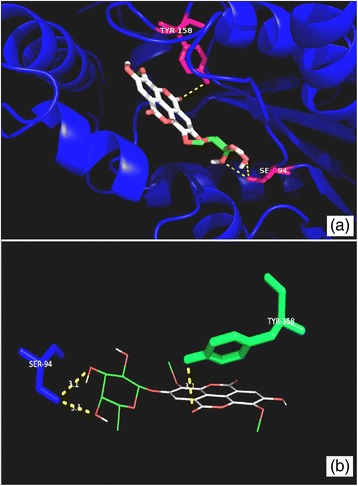
Molecular interactions between compound **2** and InhA. **a** Docked pose of **2** in the InhA binding site. The residues which interact with **2** are marked in a *hot pink colour*. **b** Interactions between **2** and InhA with the H-bond distances generated by PyMOL. *Dashed lines* represent the H-bonds

**Fig. 4:**
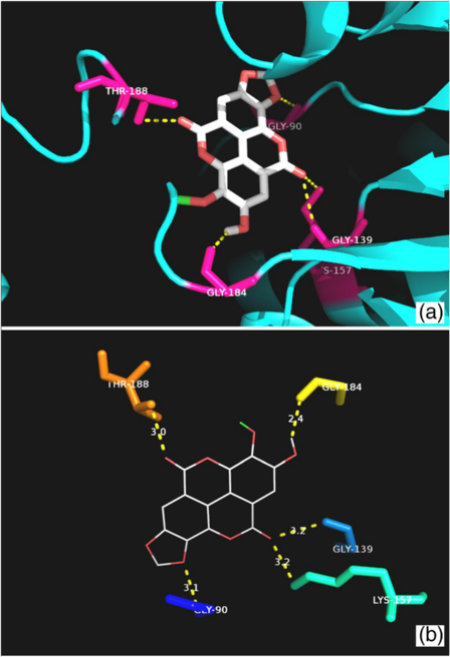
Molecular interactions between compound **1** and MabA. **a** Docked pose of **1** in the MabA binding site. The residues which interact with **1** are marked in a *hot pink colour*. **b** Interactions between **1** and MabA with the H-bond distances generated by PyMOL. *Dashed lines* represent the H-bonds

**Fig. 5:**
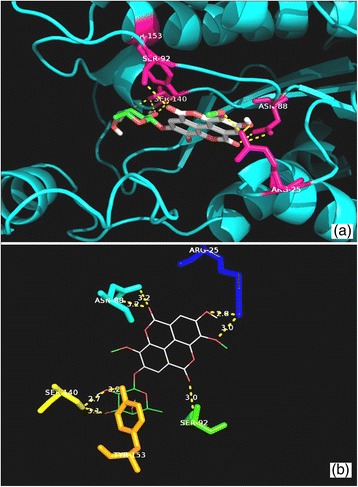
Molecular interactions between compound **2** and MabA. **a** Docked pose of **2** in the MabA binding site. The residues which interact with **2** are marked in a *hot pink colour*. **b** Interactions between **2** and MabA with the H-bond distances generated by PyMOL. *Dashed lines* represent the H-bonds

**Fig. 6:**
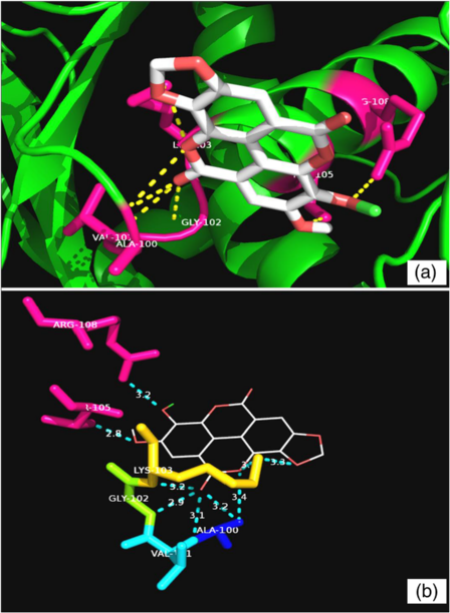
Molecular interactions between compound **1** and PanK. **a** Docked pose of **1** in the PanK binding site. The residues which interact with **1** are marked in a *hot pink colour*. **b** Interactions between **1** and PanK with the H-bond distances generated by PyMOL. *Dashed lines* represent the H-bonds

**Fig. 7:**
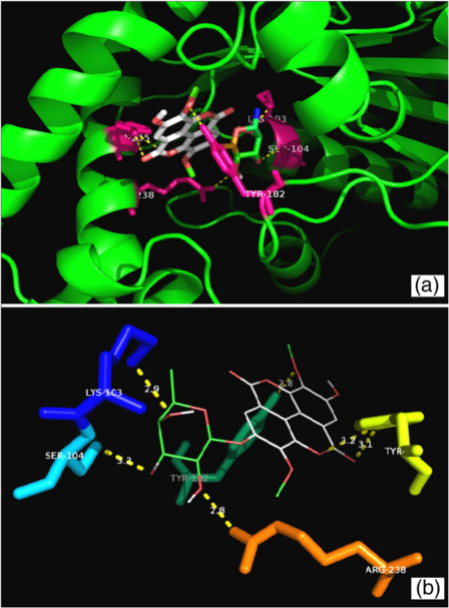
Molecular interactions between compound **2** and PanK. **a** Docked pose of **2** in the PanK binding site. The residues which interact with **2** are marked in a *hot pink colour*. **b** Interactions between **2** and PanK with the H-bond distances generated by PyMOL. *Dashed lines* represent the H-bonds

The structure of compound **2** was established following comparison of its physicochemical and spectroscopic data with those previously reported (Kang et al. 2008) (Fig. 1). Compounds **1** and **2** were selected for molecular docking simulations because, unlike other ellagic acid derivatives (3,4,3′-tri-*O*-methyl ellagic acid, 3,3′-di-*O*-methyl ellagic acid and 3-*O*-methyl ellagic acid 4′-*O*-*α* rhamnopyranoside), they had showed some activity in vitro against *Mycobacterium aurum* (Shilpi 2009). The latter is often used as a surrogate to *M. tuberculosis* in screening assays because it shares a high level of similarity with *M. tuberculosis* in its mycolic acid biosynthetic pathways and in the standard drugs used to inhibit this biosynthesis (Gupta et al. 2009). This prompted us to further investigate the potential of **1** and **2** to interact with selected enzymes that were essential to *M. tuberculosis*. The docked poses for each of the compounds were evaluated and the pose with the lowest binding free energy and the least root mean square deviation was thereby chosen (Additional file 1: Figure S1). The hydrogen bond interactions between the active compounds and selected amino acid residues for each of the target proteins are illustrated in Figs. 2, 3, 4, 5, 6 and 7. The molecular docking scores were calculated as the predicted binding free energies in kcal/mol (Table 2). The lowest binding free energy (i.e. best docking score) indicated the highest ligand/protein affinity. The *in silico* study was done in comparison with control compounds (i.e. known inhibitors of the target enzymes). The controls were isonicotinic-acyl-NADH for both InhA and MabA and a recently identified triazole-derived compound for PanK (Bjorkelid et al. 2013).

**Table 2:** Predicted binding free energies (docking scores) and detailed interactions observed between compounds **1**, **2** and the target enzymes

Test compound	Protein name	Predicted binding energy (kcal/mol)	Interaction with amino acid residues
**1**	InhA	−8.4	Ile95, Gly96, Lys165, Thr196.
	MabA	−9.4	Gly90, Gly139, Lys157, Gly184, Thr188.
	PanK	−9.7	Ala100, Val101, Gly102, Lys103, Thr105, Arg108.
**2**	InhA	−7.8	Ser94, Tyr158.
	MabA	−10.8	Arg25, Ser92, Asn88, Ser140, Tyr153.
	PanK	−11.3	Lys103, Ser104, Tyr182, Tyr123, Arg238.
Isonicotinic-acyl-NADH (control)	InhA	−11.7	Gly14, Ser20, Ile21, Lys165, Thr196.
	MabA	−9.5	Gly22, Asn24, Arg25, Ile27, Asp61, Asn88, Tyr153, Lys157, Thr188, Thr191.
Triazole-derived compound (control)	PanK	−10.6	Tyr235.

The predicted binding free energies observed for compound **1** and **2** with InhA were −8.4 and −7.8 kcal/mol, respectively. The binding free energy observed for isonicotinic-acyl-NADH against InhA was −11.7 kcal/mol. Compound **1** was found to establish hydrogen bonds with Ile 95, Gly96, Lys165 and Thr196 (Fig. 2). Isonicotinic-acyl-NADH interacted with Gly14, Ser20, Ile21, Lys165 and Thr196 residues of InhA. The hydroxyl group of the Thr196 residue has been described as a critical component of the substrate binding loop of InhA as, in association with NAD, it helps to fix the substrate on to its active site (Rozwarski et al. 1999). Compound **2** interacted with Ser94 and Tyr158 residues of InhA (Fig. 3). The X-ray crystallographic data published for PDB ID: 1BVR shows that the hydroxyl group of the Tyr158 residue is involved *via* hydrogen bonding in the interaction between InhA and its natural substrate (Rozwarski et al. 1999). In addition, the Ser94 residue plays a crucial role in the interaction between InhA and the isonicotinic-acyl-NADH complex. A mutation in the Ser94 residue to Ala94 causes *Mycobacterium* to become resistant to isoniazid (Rozwarski et al. 1998, 1999).

The binding free energies obtained for compounds **1** and **2** with MabA were −9.4 and −10.8 kcal/mol, respectively. The binding free energy of compound **1** with MabA was comparable with the control inhibitor isonicotinic-acyl-NADH (−9.5 kcal/mol). Compound **1** established hydrogen bonds with the amino acid residues Gly90, Gly139, Gly184, Lys157 and Thr188, while compound **2** interacted with Arg25, Asn88, Ser92, Ser140 and Tyr153 (Figs. 4 and 5). Isonicotinic-acyl-NADH interacted with Gly22, Asn24, Arg25, Ile27, Asp61, Asn88, Tyr153, Lys157, Thr188 and Thr191 residues of MabA. Among these amino acids, the Ser140, Tyr153 and Lys157 residues are those associated with the catalytic triad of MabA. The side chain of Tyr153 has a central role in the acid–base catalysis performed by this enzyme (Kavanagh et al. 2008). Any mutation in the Ser140 residue causes a loss of enzyme activity (Rosado et al. 2012). The amino acid Gly90 has been shown to be involved in the complexation of MabA with its natural NADPH cofactor while any mutation of Gly139 into Ala139 causes complete protein inactivation by freezing the catalytic triad into a closed form (Poncet-Montange et al. 2007; Rosado et al. 2012).

The predicted binding energies for compounds **1** and **2** with PanK (type I) were −9.7 and −11.3 kcal/mol, respectively. The triazole-derived control inhibitor showed a binding energy of −10.6 kcal/mol towards PanK. Compound **1** established hydrogen bonds with the amino acid residues Ala100, Val101, Gly102, Lys103, Thr105 and Arg108 whereas compound **2** interacted with Lys103, Ser104, Tyr123, Tyr182 and Arg238 (Figs. 6 and 7). The triazole-derived control inhibitor displayed an interaction only with the Tyr135 residue of PanK. The Ala100 to Ser104 residues are known to be part of the PanK P-loop which is responsible for the holding of ATP during catalysis (Cheek et al. 2002; Bjorkelid et al. 2013). The Arg238 acts as a connecting residue between the phosphorylated pantothenate and ATP, thereby aiding catalysis (Chetnani et al. 2010).

## Conclusions

Knowledge on the molecular interactions of natural products with essential *M. tuberculosis* targets is a potentially useful tool for the design and development of new anti-TB drugs. This *in silico* study revealed that two plant-derived compounds had the potential to interact with selected enzymes that were essential to *M. tuberculosis*. One of them, identified as pteleoellagic acid (**1**) had a docking score to MabA comparable to the control inhibitory substrate for this enzyme. Further work is required to gain a better insight into structure-active site relationships using a wider variety of structurally-related derivatives as well as to correlate the results of the docking study with in vitro enzymatic experiments in the search for new anti-TB drugs.

## Additional file

Additional file 1: Figure S1. Docked complexes of compound **1** and compound **2** at the protein binding sites. Docking of **1** (a) and **2** (b) with InhA. Docking of **1** (c) and **2** (d) with MabA. Docking of **1** (e) and **2** (f) with PanK. Surfaces represent the protein while sticks represent the active compounds. Different surface colours were chosen to represent different proteins. (JPG 306 kb)

## Abbreviations

InhA: 2-*trans*-enoyl-ACP reductase
MabA: β-ketoacyl-ACP reductase
MDR: Multi-drug resistant
NADH: Nicotinamide adenine dinucleotide (reduced form)
PanK: Pantothenate kinase
PDB: Protein data bank
TB: Tuberculosis
XDR: Extensively-drug resistant
