# Supplementary figures and images for: Molecular docking studies on InhA, MabA and PanK enzymes from Mycobacterium tuberculosis of ellagic acid derivatives from Ludwigia adscendens and Trewia nudiflora
Source: In Silico Pharmacol. 2015 Dec 8;3:10. doi: 10.1186/s40203-015-0014-1 (PMC4671986; doi:10.1186/s40203-015-0014-1)

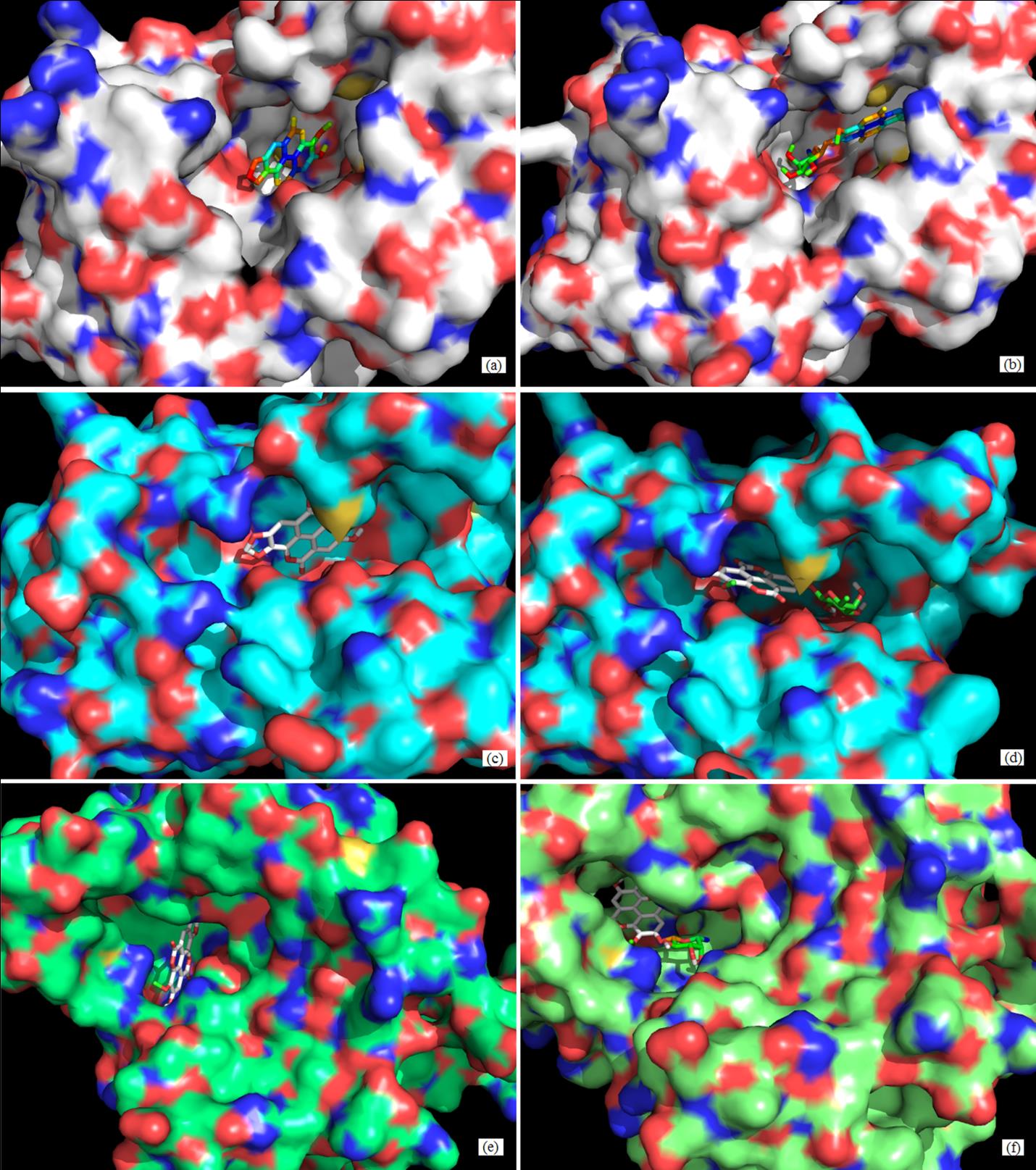

Supplement: Additional file 1: Figure S1. — Docked complexes of compound 1 and compound 2 at the protein binding sites. Docking of 1 (a) and 2 (b) with InhA. Docking of 1 (c) and 2 (d) with MabA. Docking of 1 (e) and 2 (f) with PanK. Surfaces represent the protein while sticks represent the active compounds. Different surface colours were chosen to represent different proteins. (JPG 306 kb) [file 40203_2015_14_MOESM1_ESM.jpg]
